# Nanotherapy therapy for acute respiratory distress syndrome: a review

**DOI:** 10.3389/fmed.2024.1492007

**Published:** 2024-12-06

**Authors:** Yilai Yu, Liping Qiu

**Affiliations:** Haining People’s Hospital, Haining Branch, The First Affiliated Hospital, Zhejiang University, Haining, Zhejiang, China

**Keywords:** acute respiratory distress syndrome, nanocarriers, drug delivery, lipid nanoparticles, polymer nanoparticles, inorganic nanoparticles

## Abstract

Acute respiratory distress syndrome (ARDS) is a complex and life-threatening disease characterized by severe respiratory failure. The lethality of ARDS remains alarmingly high, especially with the persistent ravages of coronavirus disease 2019 (COVID-19) in recent years. ARDS is one of the major complications of neocoronavirus pneumonia and the leading cause of death in infected patients. The large-scale outbreak of COVID-19 has greatly increased the incidence and mortality of ARDS. Despite advancements in our understanding of the causes and mechanisms of ARDS, the current clinical practice is still limited to the use of supportive medications to alleviate its progression. However, there remains a pressing need for effective therapeutic drugs to combat this devastating disease. In this comprehensive review, we discuss the commonly used therapeutic drugs for ARDS, including steroids, vitamin C, targeted inhibitors, and heparin. While these medications have shown some promise in managing ARDS, there is still a significant gap in the availability of definitive treatments. Moreover, we highlight the potential of nanocarrier delivery systems, such as liposomes, lipid nanoparticles, polymer nanoparticles, and inorganic nanoparticles, as promising therapeutic approaches for ARDS in the future. These innovative delivery systems have demonstrated encouraging results in early clinical trials and offer the potential for more targeted and effective treatment options. Despite the promising early results, further clinical trials are necessary to fully assess the efficacy and safety of nanotherapies for ARDS. Additionally, more in-depth research should be conducted to focus on the continuous development of precision therapies targeting different stages of ARDS development or different triggers. This will provide more ideas and rationale for the treatment of ARDS and ultimately lead to better patient outcomes.

## 1 Introduction

Acute respiratory distress syndrome (ARDS) is an acute respiratory disease characterized by bilateral opacities on chest imaging and severe hypoxemia caused by non-cardiogenic pulmonary edema (1). ARDS is often considered to occur as a complication of critical illness and can be induced by a variety of causes, which can be roughly divided into infectious and non-infectious causes. Pulmonary and extrapulmonary sepsis caused by pathogen infection is an important cause of ARDS, with a mortality rate of 30–40% caused by sepsis (2, 3). Aspiration of gastric contents, multiple transfusions, severe trauma with shock, non-lung protective ventilation, and pancreatitis are common non-infectious causes of ARDS, and these triggers can directly lead to ARDS through pulmonary inflammation, or indirectly through systemic inflammation and injury mediators (4, 5). The massive outbreak and ongoing progression of COVID-19 in 2019 resulted in millions of deaths worldwide, and ARDS is one of the major complications of COVID-19 and the leading cause of death in infected patients. Although we have made great strides in our understanding of the triggering causes and pathogenesis of ARDS, as well as advances in supportive therapeutic care (e.g., extracorporeal membrane oxygenation, lung-protective ventilation), there are no reliable and effective pharmacological therapies for ARDS in clinical practice, and morbidity and mortality rates are still unacceptably high. A study that surveyed 21 hospitals in Washington, D.C., USA, found that for adults, the hospitalization mortality rate for mild ARDS patients is 34.9%, for moderate ARDS patients it is 40%, and for severe ARDS patients it is as high as 46.1%, posing a serious burden on patients’ lives (6). Summarizing 29 pediatric systematic evaluations and a cross-sectional study of 145 pediatric intensive care units, it was found that, most of whom develop ARDS within 5 years of age, the incidence of ARDS is 2.3 ∼ 3% and the mortality rate is 17 ∼ 33% (7, 8), and the quality of life of the survivors is also poor. Therefore, there is a need to explore effective therapies for ARDS. In this review, we present the existing therapies such as corticosteroids, vitamin C, signaling pathway inhibitors, heparin that have been developed for ARDS as well as nanotherapy (lipid nanocarriers, polymer nanocarrier, inorganic nanoparticles) and discuss the possible problems and challenges.

## 2 Current drug therapy for ARDS

Corticosteroids are considered an important treatment for ARDS because of their ability to suppress lung inflammation by inhibiting the expression of inflammatory factors (9). Studies have shown that patients treated with glucocorticoids have a higher survival rate and reduced duration of mechanical ventilation (10, 11). Glucocorticoids, the star drugs in the treatment of ARDS, mainly include dexamethasone (DEX), hydrocortisone and methylprednisolone. DEX has been used with good results in clinical treatment. In a multicenter randomized controlled trial in intensive care units in Spain, eligible patients were randomly assigned based on balanced treatment assignments with a computerized randomization allocation sequence using blocks of 10 opaque, sealed envelopes to receive immediate treatment with dexamethasone or continued routine intensive care (control group). The number of ventilator-free days was higher in the DEX group than in the control group (between-group difference 4.8 days [95% CI 2.57–7.03]; *p* < 0.0001), and the mortality rate was significantly lower (21%) than in the control group (36%) (between-group difference −15.3% [−25.9 to −4.9]; *p* = 0.0047), demonstrating a beneficial effect in the treatment of ARDS (11). However, the clinical efficacy of corticosteroids in the treatment of ARDS has been controversial. A retrospective study by Lamouche-Wilquin et al. found no difference in mortality between dexamethasone (DEX)-treated patients in the intensive care unit of COVID-19-ARDS and the control group (12). It is hypothesized that this may be due to the fact that most of the patients in the ICU are in the advanced stages of ARDS, have a weakened immune system, and concomitant multiple underlying diseases, while the SARS-CoV-2 virus exacerbates lung injury. Therefore, there is some uncertainty about the efficacy of corticosteroids as a supportive drug, which may be less effective in critically ill patients or those with a weakened immune system. Therefore, DEX may be more suitable for clinical application as a therapeutic agent for early ARDS. In addition, a study evaluated the use of corticosteroids in patients with COVID-19-related ARDS and found that methylprednisolone use may improve outcomes in some patients, but also delay the clearance of the virus (13). This shows that treatment needs to comprehensively consider the patient’s condition characteristics. And a meta-analysis showed that systemic corticosteroid treatment was associated with reduced mortality in critically ill patients with COVID-19 (14). Analysis pointed out that this type of treatment can effectively relieve inflammation, but it may pose certain risks and needs to be applied cautiously in clinical practice.

The antioxidant effect of ascorbic acid was highlighted in the treatment of COVID-19, a patient with COVID-19-ARDS who had extremely low plasma levels of vitamin C due to compensatory overdose of vitamin C by the immune system (15). A randomized controlled study in Iran showed a linear correlation with higher survival in COVID-19 patients with supplemental intake of vitamin C (16). Although vitamin C does have these benefits in the treatment of ARDS, it is more commonly used clinically as a supplement in combination with other drugs to enhance the efficacy of other drugs such as glycyrrhizinic acid and NAC through an additive effect (17, 18). Therefore, vitamin C is considered a supplementary treatment rather than a specific medication for ARDS. Intravenous low to moderate doses of intravenous vitamin C do not relieve patients’ clinical symptoms, and there is no significant difference in C protein levels (19, 20). The inability of vitamin C alone to alleviate inflammation and, more importantly, the inability to continuously monitor therapy without knowing the patient’s initial vitamin C level on admission limits the clinical use of this drug.

The development of ARDS usually involves multiple signaling pathways, therefore, some signaling pathway inhibitors are also useful in alleviating ARDS. Several JAK inhibitors such as ruxolitinib, baricitinib, fedratinib, tofacitinib have been approved by the European Medical Association. They reduce inflammation and improve prognosis in severe ARDS. Unfortunately, the efficacy of JAK inhibitors is influenced by the time of initiation, and the use of JAK inhibitors increases the incidence of anemia and thrombocytopenia, promotes autoimmunity, and promotes thrombus formation (21). Remdesivir, aclatinib, and infliximab have shown encouraging results in the fight against COVID-19. Remdesivir, a nucleoside analog, significantly inhibits cytokine storm damage through inhibition of the dsdna-associated NF-κB pathway, reducing patient mortality and adverse event rates (22, 23). Of course, the side effects of diarrhea, nausea, and vomiting are unavoidable (24). Activation of NLRP3 inflammatory vesicles plays a key role in the outbreak of cytokine storms. anakinra, an inhibitor of NLRP3 inflammatory vesicles, reduces the need for mechanical ventilation and mortality in patients (25). However, low doses of anakinra were not effective in patients with severe ARDS, with no significant improvement in mortality (29% vs 46%, *P* = 0.08) (26).

Heparin, a glycosaminoglycan anticoagulant, is widely used in clinical practice. Heparin is used in the short-term management of ARDS to prevent thromboembolic complications, especially in critically ill patients Preclinical trials have shown that nebulized heparin reduces microvascular thrombosis and promotes coagulation activation, reduces levels of inflammatory markers, reduces ventilator days, and improves functional scores (27, 28). Unfortunately, the clinical mode of administration of heparin is nebulization, during which patients have an increased risk factor for bleeding at the tracheotomy site or hemoptysis due to a transient increase in airway pressure (28). Therefore, further validation regarding the efficacy and safety of heparin in the treatment of ARDS remains to be done in additional clinical trials.

In addition, antibiotics, neuromuscular blockers, and inhaled vasodilators can be used for ARDS management in the short term. They play different roles:antibiotics are used to treat or prevent bacterial infections that can complicate ARDS. Neuromuscular blockers (e.g., rocuronium) facilitate mechanical ventilation by reducing muscle movement, thereby enhancing ventilation. And inhaled vasodilators can improve oxygenation by dilating pulmonary blood vessels, although their use is more limited and context-dependent.

Considering that the limited efficacy of currently used drugs, we are pinning our hopes of treating ARDS more on the discovery of emerging drugs. We are focusing our attention on nanodelivery drug systems and mesenchymal stem cell (MSC) therapies, which may have great potential to cure ARDS patients.

## 3 Emerging therapies for ARDS

### 3.1 Nanodelivery systems

So far, most of the drugs for treating ARDS are only at a supportive level and cannot guarantee a complete cure. This is because the free drug cannot reach the required dose at the target organ after blood circulation, and the efficacy is greatly reduced. Besides, the non-targeting nature of the drug may cause side effects to other organs. In recent years, the scientific community has been greatly encouraged by the favorable therapeutic efficacy and safety demonstrated by nanomedicine-based delivery systems. Nanocarrier-based drug delivery system consist of 10–1000 nm drug and carrier materials, in which the nanodrugs include liposomes, extracellular vesicles, and polymeric micelles (29). The encapsulation of nanocarriers can effectively prevent the drug from being degraded, increase the concentration and activity of the drug, and achieve precise release in the lungs, while avoiding undesirable side effects. The delivery of NPs to the lungs and their distribution and deposition are related to many factors, including respiratory rate, lung volume, airflow, particle size and zeta potential (30, 31). Small particles (1–5 μm) are predominantly deposited in the lungs, while larger particles (>10 μm) are predominantly deposited in the pharyngo-oral region. NPs currently loaded with antibiotics, peptides and nucleic acids have been shown to target macrophages, neutrophils, dendritic cells and B cells to suppress inflammation (32). A variety of nanoparticles have been developed for the treatment of lung injury, especially lipid nanocarriers and polymers. They offer unique advantages in the treatment of ARDS, such as biocompatibility, stability, controlled drug release and degradation (33, 34).

#### 3.1.1 Lipid nanocarriers

Liposomes usually consist of a lipid bilayer consisting of single or multiple concentric lipid bilayers and aqueous compartments. The hydrophilic drug is localized in the aqueous phase while the lipophilic agent is embedded in the phospholipid bilayer, which ensures good affinity and compatibility of the liposome with the cell membrane. Liposomal drugs are usually fused to the cell membrane and release the drug into the cell or enter the cell by encapsulation. Therefore, liposome-loaded drugs can improve drug efficacy by altering pharmacokinetics and enhancing drug activity. Currently, liposomes have been developed as a more mature drug delivery system in nanomedicine, especially showing great potential in the treatment of ARDS.

In an animal model showing ARDS pathology, DPPC liposomes encapsulating methylprednisolone (MPS), and NAC were loaded with efficiencies of 98 and 92%, which significantly reduced the levels of IL-6, IL-1β, and TNF-α in the BALF, and alleviated the inflammation and mucus secretion of the lungs (35). To improve the targeted delivery of liposomes, scientists have developed various ligands such as antibodies, peptides, mannosylation, and nucleic acids to modify liposomes as a way to increase the therapeutic index of drugs and reduce the adverse drug reactions. The investigators prepared a liposome-encapsulated active substance, Nimbolide (iRGD-NIMLip), conjugated to the cyclic peptide iRGD and evaluated its pharmacological activity. The iRGD-NIMLip had recognition specificity and were able to target the lungs efficiently, thus, the lungs in the iRGD-NIMLip treatment group exhibited more NIM accumulation and showed higher anti-inflammatory activity compared to DEX (36). Macrophages have specific receptors for L-arginine on their surface, which enables L-arginine-functionalized liposomes encapsulating curcumin (Cur) to target M1 macrophages with greater precision and increasing the accumulation of Cur in the lungs (37). Mannosylated liposomal ciprofloxacin showed better targeting and increased lung drug release (38). Functionalization of DEX-loaded immunoliposomes (SPA-DEX-NLPs) with a lung surface-active protein A (SP-A) antibody resulted in release of DEX 40-fold more efficiently than conventional direct DEX injections, significantly enhancing drug targeting efficiency (39). Inflamed lungs recruit large numbers of neutrophils. Taking advantage of this feature, αFGF@NMLs were constructed by encapsulating neutrophil membranes on αFGF-loaded liposomes to treat lung-injured mice, which optimized the distribution of αFGF in the lungs and elevated the concentration and residence time at the lesions (40). Coupling ICAM-1 antibody to lipid nanocarriers conferred the ability to actively target lung endothelium (41). Inflammatory sites of infection present an acidic microenvironment, so by designing drugs that sense the pH of the infection site is an effective way to increase the targeting of nanodrugs. Luo et al. obtained multifunctional hybrid nanoparticles, Spe@HNPs, by modifying liposomes loaded with dagamycin using a pH-sensitive polymer, poly(β-amino ester) (PBAE) (42). Spe@HNPs have a high degree of pH sensitivity and stability, which implies that Spe@HNPs have longer *in vivo* circulation time and pH-triggered drug release, and increase the rate of drug release and cumulative release. In addition, the positive charge on the surface of PBAE disrupts the bacterial cell wall and induces bacterial death, thereby alleviating bacteria-induced ARDS. A recent study demonstrated an inhalable biomimetic nanosystem (D-SEL) with the modifying enzyme DNase I affixed to exosomes and liposomal hybrid nanocarriers, which enables localized and sustained release of DNase I to inhibit neutrophil activation and promote macrophage M2 polarization, remodeling lung immune homeostasis (43) (Table 1).

**TABLE 1:** Summary of lipid nanocarriers drugs modified with different ligands for the treatment of lung injury.

Ligand type	Lipid nanocarriers	Size (nm)	Charge (mV)	Embedding efficiency	Dose (route) *in vivo*	Animal model (route)/cell line	Treatment outcome	References
Polypeptide	iRGD-NIMLip	171.5 ± 25.05	−21.42 ± 0.78	85.24% ± 0.97%	3 mg/kg (i.p. 50 h pre)	Male C57BL/6 mice-LPS (i.p.)/BEAS-2B and RAW 264.7	Body weight lung weight index↑; inflammatory cell↓; IL-1β, IL-6, IL-17α, IL-2, TNF-α, TGF-β↓; H&E, TB↓; ROS↓	(36)
Polypeptide	Arg-Cur-Lip	142.73 ± 13.47	−24.67 ± 0.56	103.27 ± 3.86%	1 mg/kg (i.nh. 4, 12, 24 h post)	Male Wistar rats-LPS (inhal.)/RAW264.7	Macrophage targeting, M1 polarization↑; TNF-α, NO, IL-1β, IL-6↓; H&E↓	(37)
Polypeptide	GALA-MEND	Non	+ 15 ± 2	Non	1 mg/kg siRNA (i.v.)	ICR mice/A549	Lung targeting↑; cellular uptake↑	(44)
Mannosylation	Mannosylated CPFX-liposomes	1005 ± 101	−70	Non	250 μL/kg (inhal.)	Male SD rats/NR8383	Uptake rate↑; lung targeting↑; antibacterial effect↑	(38)
Mannosylation	Man-cationic liposome/FAM-labeled NFκB decoy complexes	100	+ 60	Non	50 μg (i.t. 0.5 h pre)	Male Wistar rats-LPS (i.t.)	H&E↓; MPO↓; Macrophage targeting↑; TNF-α, IL-1β, CINC-1↓; neutrophils↓	(45)
Mannosylation	DPML	110 ± 6.9	0.77 ± 0.22	99 ± 1.1%	0.5 mg/kg (i.t. 3, 24 h post)	Male Wistar rats-LPS (i.t.)	H&E↓; MPO↓; Macrophage targeting↑; TNF-α, IL-1β, CINC-1↓; neutrophils↓	(46)
Mannosylation	miR-146a-MLNP	218.7 ± 13.2	24.2 ± 4.7	Non	0.1 nmol (i.t.)	Male C57BL/6 mice-hemorrhagic shock/AMs and Pneumocytes	O_2_ saturation↑; Macrophage uptake↑; BAL protein↓; CXCL1/KC, MIP-1α, IL-8, IL-6↓	(47)
Nanovesicle	TPCA@LNVs	183.8 ± 9.0	+ 38	63 ± 6.7%	0.5 mg/kg (i.v. 1, 4, 24, 48 h post)	Male C57BL/6 mice-LPS (i.t.)/RAW264.7, MLE-12 and HUVEC	H&E↓; W/D↓; BAL protein↓; lung targeting↑; macrophages, neutrophils↓; ROS, MPO, MDA↓; NO, TNF-α, IL-1β, IL-6↓	(48)
Antibody	SPA-DXM-NLP	136 ± 38	Non	92 ± 3%	2 mg/kg (i.v. 0.25, 0.5, 1, 2, 4, 8, 12 h post)	Male SD rats-bleomycin (i.t.)	Survival rate↑; H&E↓; lung targeting↑; TNF-α, TGF-β1↓	(39)
Antibody	MPS-NSSLs-SPANb	106	Non	92.5 ± 0.5%	MPS dose at 0.5 mg/kg (i.v.)	Male SD rats-bleomycin (i.t.)	H&E↓; lung targeting↑; TNF-α, IL-8, TGF-β1↓	(49)
Antibody	ICAM/DEX/NLCs	235.9 ± 1.8	37.4 ± 0.7	82.93 ± 0.94%	1.2 mg/kg (i.v.)	Male Balb/c mice-LPS (i.t.)/EAhy926	H&E↓; cell viability↑; lung targeting↑; neutrophils↓; TNF-α, IL-6↓	(50)
Antibody	anti-ICAM/SV/NLCs	354.7 ± 18.2	−32.1 ± 3.2	96.78 ± 0.12%	2 mg/kg (i.v.)	Male Balb/c mice-LPS (i.t.)/EAhy926	H&E↓; lung targeting↑; TNF-α, IL-6↓	(51)
Neutrophil membrane	aFGF@NMLs	107.4 ± 2.7	−12.2 ± 1.9	80%	1 mg/kg (i.v.)	Male C57BL/6 mice-LPS (i.t.)/BEAS-2B and RAW 264.7	MDA↓; SOD↑; ROS↓; cell viability↑; CD68, IL-6, IL-1β, TNF-α, HMGB-1↓; lung targeting↑; H&E, TUNEL↓; total protein↓; W/D↓;	(40)
Live NEs	Lip@MH	123.57 ± 0.71	−18.73 ± 0.8	98.2 5 ± 0.31%	3 mg/kg (i.v.)	Male C57BL/6 mice-P.aeruginosa infection/RAW 264.7 and A549	W/D↓; lung targeting↑; TNF-α, IL-6, MCP-1, NO, IFN-β↓	(52)
Polymer compounds	Spe@HNPs	198	−55	90%	4 mg/kg (i.v.)	CD-1 mice- MRSA BAA40 (intratracheal administration)	Total protein↓; TNF-α, IL-6, IL-1β↓; lung weight↓; leukocyte↓; CFU↓	(42)
Enzyme	MPS/D-SEL	342.2 ± 5.6	−12.1 ± 0.6	90%	50 μg/mL (inhal.)	C57BL/6 mice-LPS (intratracheal administration)/HBEC, A549, and AMs	H&E↓; Macrophage targeting and M2 polarization↑; neutrophil↓; TNF-α, IL-6, IL-1β↓; MPO↓	(43)
Enzyme	DNase-I pMNSs	170	−10.9	Non	100 units (i.v. post)	Male C18 BL/20 mice-Septic CLP	MPO↓; neutrophil↓; IL-1β, IL-6, IFN-γ, TNF-α↓	(53)

The development of ARDS often presents multiple pathological features, such as an acidic microenvironment at the site of inflammation, endothelial cell dysfunction, cytokine storm, and imbalance of immune homeostasis including macrophages and neutrophils (1, 42, 54–56). Based on these pathological features, it is possible to modulate the physicochemical properties of the delivery system and design specific ligands to modify the liposomes encapsulating the therapeutic drug for multifunctionalization of the delivery vehicle. The modified ligands will either have a therapeutic effect to achieve bi-drug therapy or will be able to modulate drug release from liposomes to improve pharmacokinetics. In any case, drug delivery systems that modify liposomes with specific ligands can achieve precise targeting, improve therapeutic index and reduce adverse side effects, and are promising therapies for the treatment of ARDS in the future.

In addition to carrying drugs for precise targeting to the lungs, liposomes can enhance drug penetration and retention by carrying nucleic acids, thereby increasing the bioavailability of therapeutic drugs or genes (57). The complexes formed by liposomes and nucleic acids can deliver a variety of target genes, including small interfering RNA (siRNA), micro (miRNA) for therapeutic purposes. siRNA therapeutics have been widely used in the treatment of a variety of diseases, but the delivery effect is greatly reduced due to its poor stability and the obstruction of the intra- and extracellular barriers. siRNAs have a negative charge on their own, and due to electrostatic interactions, siRNAs are able to bind to cationic liposomes to form stabilized lipid complexes to protect nucleic acids from degradation by intra- and extracellular enzymes and to enhance their intracellular release. siRNA-liposome complexes enhance the safety of siRNAs and precisely target lungs to regulate gene expression. BRD4 siRNA was complexed with DOTAP to form a 96.24 ± 18.01 nm stable lipid complex (BRD4-siRNA-LP). After the nanomedicine carrying BRD4 siRNA reaches the lungs, it is taken up by the cells through endocytosis and releases the BRD4 siRNA, which inhibits the nuclear translocation of STAT3 and reduces neutrophil infiltration and mast cell accumulation (58). Folate-modified liposomes delivering siIRF5 similarly showed anti-inflammatory effects (59). After intratracheal administration of cationic liposomes loaded with receptor-interacting protein 2 (Rip2) in ALI model mice, knockdown of Rip2 successfully ameliorated lung inflammation compared to controls (60). In contrast, neutral liposomes, such as cholesterol and DOPC, rely heavily on encapsulating siRNA within liposomes, which appears to enhance siRNA safety but may also affect siRNA capture rates (61). Moreover, there are novel cationic liposomes, stabilized nucleic acid lipid particles (SNALP), which overcome the intracellular barrier of siRNA and prevent degradation (Table 1).

It is worth noting that liposome physicochemical properties correlate with therapeutic efficacy. Smaller sizes are more lipophilic (62) and more efficiently released, whereas larger sizes have greater lung-targeting capacity and intracellular internalization (50). Cations seem to show stronger anti-inflammatory effects, and anionic liposomes have stronger cellular uptake and wider distribution (50, 63, 64). However, despite these advantages, the use of cationic liposomes raises safety concerns and must be carefully managed in clinical applications. Due to their positive surface charge, they easily interact with biological membranes and serum proteins, which can trigger immune responses such as inflammation and hemolysis, as well as accumulate in the liver and spleen, impairing organ function. Moreover, these liposomes may induce excessive inflammatory responses by interacting with Toll-like receptors, potentially causing cytotoxicity and organ damage. To mitigate these risks, polyethylene glycol (PEG) is often added to the liposome surface to reduce immune recognition, although this may also compromise drug delivery efficiency. The shape, hydrophilicity, and surface modification of liposomes affect immune properties and efficacy in different ways. Further understanding of the physicochemical parameters of nanoparticles will help us in designing more feasible nanomedicines. According to the pathological characteristics of ARDS, we should select effective delivery drugs and suitable ligands, and adjust the physicochemical properties of liposomes to improve their solubility and cellular uptake, so as to make them more widely distributed in the inflammation site and ensure the therapeutic effect. Currently, intravenous (IV) administration is the main route of drug delivery for lipid delivery systems. Unfortunately, IV administration is readily absorbed by the liver and spleen after circulation, and less of the drug reaches the lungs, resulting in a significant reduction in efficacy (65, 66). Pulmonary drug delivery, on the other hand, can deliver drugs directly to the site of inflammation, thus avoiding the first-pass effect of drugs due to body circulation, reducing enzymatic degradation and effectively increasing drug concentrations in the lungs (67). In the future, researchers should aim to study more drugs based on nanodelivery systems that can be delivered via the lungs, bringing hope to the treatment of ARDS.

Lipid nanoparticles (LPN) are one of the most successful nanodelivery carriers, which are capable of delivering antibiotics, nucleic acids to target organs. LPN are generally composed of four lipids including (1) ionizable cationic lipids, (2) 1,2-distearoyl-sn-glycero-3-phosphorylcholine (DSPC), (3) cholesterol, and (4) polyethylene glycol (PEG) lipid couplings, which help in the efficient encapsulation of nucleic acids in LPNs, cellular uptake for internalization, and endosomal escape of nucleic acids (68). Currently, LPN have made a major breakthrough in RNA delivery. Pfizer/BioNTech utilized lipid nanoparticles and nucleoside-modified RNA to create a vaccine against the receptor-binding domain (RBD) of the S-protein in SARS-CoV-162 and showed 95.46% efficacy and safety in clinical phase 2 and 3 trials. mRNA-1273 manufactured by Moderna, also formulated with lipid nanoparticles, demonstrated efficacy and safety in the COVID-19 trial demonstrated potent antibody activity (69, 70). Both of these drugs are licensed and a number of other lipid nanoparticle-mRNA formulations are in the clinical evaluation phase.

#### 3.1.2 Polymer nanocarrier

In addition to lipid carriers, another common type of nanocarriers are spherical polymeric nanocarriers, which are amphiphilic spatially conformationally stable macromolecules usually composed of polyethylene glycol, polyvinyl alcohol (71). Most of them are biodegradable and biocompatible, and their high drug-carrying capacity makes them favorable carriers for drug delivery. Poly(lactic-co-glycolic acid (PLGA) is the more commonly used polymer approved by the FDA, and sialic acid-modified PEG-PLGA microspheres loaded with curcumin target ALI (72). Nanoparticles composed of surfactant protein D and PLGA showed high biological activity within days (73). Nasal delivery of hesperidin-loaded chitosan nanoparticles (HPDs/NPs) showed enhanced cellular uptake in the inflammatory microenvironment and inhibited cytokine storm syndrome in an ALI/ARDS mouse model (74). Considering the side effects of celecoxib (CXB) (headache, drowsiness, nausea), the anti-inflammatory drug CXB was encapsulated by PLGA, which exerts anti-inflammatory effects after targeting to reach the lungs and being internalized by lung macrophages. The degradable polymer particles help lung macrophages to accurately recognize and well prolong the release of CXB (75). PLGA-SV NPs were constructed by packaging simvastatin (SV) into PLGA nanoparticles, which could maximize SV uptake and inhibit the production of inflammatory factors (76). Long-term use of DEX can have some side effects on non-target organs, while DEX microspheres prepared with PLGA and DPP4 (4:1) showed satisfactory therapeutic efficacy by inhibiting inflammatory infiltration and oxidization in the lungs with high specificity and targeting efficiency (77) (Table 2). Interestingly, micelles can be further optimized by using specific ligands to enhance their cell permeability.

**TABLE 2:** Summary of polymeric nanocarriers for the treatment of lung injury.

Polymer nanocarrier	Polymeric material	Size (nm)	Charge (mV)	Dose (route) *in vivo*	Animal model (route)/cell line	Treatment outcome	References
SA/Cur-TPP/MS (MS-3)	PLGA	851 ± 31.80	–24.2 ± 2.1	4.24 mg/kg (i.v.)	Male ICR mice-LPS (i.t.)/HUVECs	Lung targeting↑; W/D↓; H&E↓; SOD↑; NO, MDA, ROS↓; TUNEL↓; TNF-α, IL-6↓; cell viability↑	(72)
SP-D-PLGA NPs	PLGA	105 ± 3	−36 ± 5	2 mg/kg (i.t.)	Male C57BL/6 mice/A549	H&E↓; Macrophages, Neutrophils↓	(73)
HPD/NP	PLGA-PEG	200	+ 22	10 mg/kg (i.n.)	C57BL/6 mice-LPS (i.p.)/A549 and HUVEC	H&E↓; EBA flux↓; W/D↓; BAL protein↓; cell viability↑; endothelial permeability↑; IL-6, IL-β, caspase1↓	(74)
PLGA-SV NPs	PLGA	231.8 ± 8.8	31.75 ± 1.5	Equivalent to 20, 10, 5 mg SV/kg (i.g.)	Male Wistar rats- paraquat (p.o.)	Body weight↑; H&E↓; IL-6, IL-1β, TNF-α↓; GSH↑	(76)
DEX-DPPC-PLGA	DPPC-PLGA	8830 ± 320	Non	Equivalent to dexamethasone 1 mg/kg B.W. (i.v.)	Male Wistar rats- LPS (i.t.)	Lung targeting↑; H&E↓; IL-6, IL-1β, TNF-α↓; Neutrophils↓	(77)
PTKNPs@Dex	PPADT-PTKU	400	–22	8.67 mg/mL (i.v.)	Male C57BL/6 mice-LPS (i.t.)	Survival rate↑; H&E↓; TUNEL↓; W/D↓; LDH, MPO↓; ROS↓; MDA↓; BAL protein↓; IL-6, IL-1β, TNF-α↓; Neutrophils↓	(78)
DPM prodrug NPs	PEI	115 ± 1	31 ± 1	2.5 mg/mL (i.v. 1, 2, 4, 12, 24 h)	Male Kunming mice-LPS (inhal.)/RAW264.7	Lung targeting↑; H&E↓; W/D↓; TNF-α↓; cell viability↑	(79)
RBC-MPSS-CSNPs	CS	233.3	+ 30.0	2 mg/kg (i.v. 0, 2, 4, 6, 8, 12, 24, 48 h)	Male F12 rat-LPS (i.p.)	Lung targeting↑; H&E↓; W/D↓; BAL protein↓; IL-6, TNF-α↓; MPO↓; IL-1β, caspase1↓	(80)
TPCA-1-NPs-anti-ICAM-1	Biotin-PEG-PAE-PEG-Biotin	100	Non	equal to 2 mg/kg of TPCA-1 (i.v.)	Adult CD-1 mice-LPS (i.n.)/HUVECs	Lung targeting↑; H&E↓; IL-6, TNF-α↓; BAL protein↓; Neutrophils↓	(81)
α-bis-LNCs	poly(ε-caprolactone)	160 ± 10	−8.1 ± 1.0	30, 50, or 100 mg/kg (i.g. pre)	Male A/J mice-LPS (i.n.)	H&E↓; Lung targeting↑; AHR↓; MPO↓; Neutrophils↓; CXCL-2, KC↓	(87)
RSV-LNCs	poly(ε-caprolactone)	241 ± 7	−14.1 ± 2.3	5 mg/kg (o.p. 1, 4, 6, 12, 24 h pre)	Male A/J mice-LPS (i.n.)	H&E↓; Neutrophils↓; BAL leukocyte↓; MPO↓; MDA↓; SOD↑; IL-6, KC, MIP-1α, MIP-2, MCP-1↓; AHR↓	(88)
miR-146a-MLNP	PEI	218.7 ± 13.2	24.2 ± 4.7	0.1 nmol (i.t.)	Male C57BL/6 mice-hemorrhagic shock/AMs and Pneumocytes	O_2_ saturation↑; Macrophage uptake↑; BAL protein↓; CXCL1/KC, MIP-1α, IL-8, IL-6↓	(47)
PLGA-PD-PEG-DNase-1 NPs	PLGA	217 ± 1.63	−12	100 units DNase-1 (i.v.)	Male C57BL/6 mice-septic CLP	Survival rate↑; H&E↓; Neutrophils↓; MPO↓; IL-6, IL-1β, IFN-γ, TNF-α↓	(83)
PONI-Guan/siRNA	PONI-Guan	170	+ 15	0.14–0.28 mg/kg (i.v.)	Male BALB/c mice-LPS (i.p.)/RAW 264.7	H&E↓; Lung targeting↑; cellular uptake↑; TNF-α↓	(89)

Polymeric nanoparticles (NPs) are important carriers for piggybacking therapeutic drugs for ARDS. Larger molar masses of polymers such as 20 to 50 KDa increase the size of the drug carrier allowing it to avoid renal clearance and can deliver low molar mass siRNAs or small molecule drugs well. Lower molar masses (<5 KDa) are better suited for delivering larger drugs such as antibodies and nanoparticle drugs (70). The PTKNPs@Dex nanoplatform assembled with poly (1,4-phenylpropanonedimethylene thioketal) (PPADT) and polythioketal urethane (PTKU) reached the lungs and rapidly degraded the NPs and released DEX, which showed more potent anti-inflammatory and antioxidant effects as compared to free DEX (78). The NPs not only delivered the drugs to the lungs but also helped to scavenge excessive ROS by its large number of thione bonds in its main chain. A pH-responsive DPM prodrug constructed from mannose co-modified branched polyethyleneimine (PEI) linked to DXM via Schiff base bonding showed enhanced lung targeting, with fewer inflammatory infiltrates and significant relief of pulmonary edema (79). The efficacy of methylprednisolone sodium (MPSS) is limited by its hormone-dependence and side effects. The RBC-MPSS-CSNPs, prepared by tight binding of chitosan NPs to erythrocytes, was able to achieve a slow release of MPSS, reduce the accumulation of MPSS in the liver and kidney and increase the concentration of MPSS in the lungs (80). Based on the low pH characteristics of the inflammatory microenvironment, the pH-sensitive hydrophobic chain segment PAE and hydrophilic chain segment PEG-biotin were assembled into polymeric micelles as a way to ensure the triggering of drug release at low pH. A large number of neutrophils will be recruited at the inflammation site, and the ICAM-1 antibody, which promotes neutrophil transport, will be encapsulated on the surface of the nanoparticles, which is more conducive to the targeting of the inflammatory sites in the lungs. Functionalized polymer micelles carry the anti-inflammatory drug TPCA-1 to the inflamed lung to treat ALI/ARDS (81) (Table 2). By designing the surface coating of nanoparticles, biocoupling between the polymers, and carrying different drugs, the polymers have multiple functions and properties. This unique design can better control the drug release and enhance the targeting of the polymer nanoparticles, which provides more ideas for us to develop new nanomedicines in the future.

Polymeric nanocarriers can also deliver nucleic acids to target organs to regulate gene expression like liposomes. Mannose-coupled nanoparticles delivered miR-146a to lung macrophages, causing miR-146a expression to exceed 10000-fold of the physiological dose, resulting in a rapid onset of action (45). Lipid nanoparticles delivering prostaglandin E synthase small interfering RNA antibody (anti-PGE2-siRNA) controlled pro-inflammatory macrophage polarization and showed potent anti-inflammatory properties (82). It is worth mentioning that polymers are good carriers for enzyme delivery. Polydopamine-poly(ethylene glycol) nanoparticulates encapsulating DNase-1 improved the half-life of DNase-1, protected DNase-1 from inactivation, and effectively reduced cfDNA levels and neutrophil activity (83) (Table 2).

Polymers indicate that active agents are commonly used to encapsulate or disperse drugs or other active ingredients in polymeric nanocarriers. This is due to the ability of PLS to (1) reduce surface tension. (2) Stabilize the dispersed state of nanoparticles so that they are uniformly dispersed in liquids and prevent agglomeration or precipitation. (3) Help control the size, shape, and surface properties of the nanocarriers for specific applications. Surfactant replacement therapy is gradually coming into the public eye. It is worth mentioning that the role of polymeric lung surfactants in mitigating lung injury is gradually being recognized. A novel polymeric lung surfactant (PLS) consisting of poly(styrene-block-ethylene glycol) (PS-PEG) block copolymer micelles has been shown to reduce the severity of lung injury when injected transpharynxally (84). There are a number of advantages over conventional lipid-based surfactants, as the highly hydrophobic and hydrophilic PEG coatings at the core of PS together form an insoluble monolayer that avoids interactions with surface-active proteins, and mice treated for 14 days showed no toxic side effects (84, 85). In addition to the advantage that PS-PEG has an extremely low surface tension under high compression, PS is usually required in high doses, whereas PLS is easier to administer by nebulization because it can be used in lower concentrations. In addition, PLS can be mass-produced at low cost compared to animal surfactants, which makes it possible for PLS to become a therapeutic agent for clinical ARDS (86).

#### 3.1.3 Inorganic nanoparticles

The main inorganic nanomedicines that have been studied more extensively include gold nanoparticles, selenium nanoparticles, and cerium dioxide nanoparticles. These inorganic nanoparticles can serve as excellent imaging agents to aid in disease diagnosis, as well as effective carriers to improve the bioavailability of nanomaterials. They typically have smaller diameters and more stable properties. Gold nanoparticles are considered effective nanocarriers in the clinic because they are readily synthesizable and can be precisely sized from a few nanometers to several hundred nanometers. It has been shown that gold nanoparticles coupled with Curcumin (Cur) attenuated the expression of inflammatory factors and the accumulation of ROS and alleviated lung inflammation (90). However, they accumulate in the liver and spleen and are not easily degraded, so researchers have proposed modifying GNP with peptides to improve their safety and efficacy. The thiol groups in peptides bind readily to gold and can alter the surface chemistry of GNPs and confer new biological activities (91). This peptide-GNP hybrid was able to drive polarization of mouse bone marrow-derived macrophages (BMDMs) toward an anti-inflammatory M2 phenotype and reduce alveolar and interstitial M1 macrophages in BALF and lung tissues to promote the regression phase of ARDS (89). Peptide-GNP hybrids inhibit the TLR signaling pathway in macrophages to attenuate lung inflammation and accumulate mainly in the lungs and intestines, with less than 3% accumulation in the liver and spleen (92). It has also been shown that p12GNP has enhanced cellular uptake and pH buffering capacity and is superior in alleviating inflammation and diffuse lung injury (93). Se, an essential trace element, is well known for its antioxidant capacity, and porous Se@SiO2 nanospheres have been demonstrated to resist ARDS by exerting an anti-inflammatory and antioxidant effect (94). The large specific surface area of the porous Se@SiO2 nanospheres and the uniform distribution of the particle sizes allow the nanospheres to release Se slowly and continuously with a good controlled-release performance, and thus the Se Se@SiO2 nanospheres may have higher biosafety and stability. Porous Se@SiO2 nanosphere treatment alleviated ROS and reduced levels of NF-κB, p-NF-κB, and IL-1β (94). Se@SiO2 nanospheres may exert therapeutic effects through multiple pathways, and mitochondria are also important targets of Se@SiO2 nanospheres. Mitochondria provide energy for cellular activity, influence cellular metabolism, and are also the primary site of reactive oxygen species production. Under oxidative stress, Se@SiO2 nanoparticles stabilize the structure of the mitochondrial membrane, maintain the normal shape of the mitochondria, and reduce mitochondrial rupture and damage. In addition, Se@SiO2 nanoparticles enhance the activity of I, III and V complexes in the mitochondrial respiratory chain. Therefore, Se@SiO2 nanospheres can target mitochondria, improve mitochondrial function, and reduce the number of inflammatory cells and neutrophils, thereby alleviating inflammatory response and diffuse lung tissue injury (95). The use of nanoparticles piggybacked with a potent anti-inflammatory material, cerium dioxide (CeO_2_), can mitigate excessive accumulation of CeO2 in liver and kidney tissues *in vivo* and accelerate drug metabolism. In the LPS-induced pneumonia model in rats, CeO2 NPs showed a strong capacity to scavenge ROS, significantly reducing ROS production in blood and lung tissue and thereby mitigating infection-induced oxidative stress. Additionally, CeO2 NPs reduced the expression of pro-inflammatory cytokines such as TNF-α, IL-6, and CxCL2, disrupting the amplification feedback loop of cytokine production typically triggered by inflammatory stimuli and showcasing notable anti-inflammatory effects. Importantly, CeO2 NPs enhanced oxygen consumption in both healthy and pneumonia-affected rats, likely due to their positive impact on mitochondrial function, stabilizing mitochondrial membrane potential and promoting ATP production, thereby improving cellular energy states under inflammatory challenge (96) (Table 3).

**TABLE 3:** Summary of inorganic nanoparticles for the treatment of lung injury.

Inorganic compound	Inorganic nanoparticles	Size (nm)	Charge (mV)	Dose (route) *in vivo*	Animal model (route)/cell line	Treatment outcome	References
Gold	GNP-Cur	20	±25	5.12 mg/L (i.n.)	Male Wistar rats- LPS (i.p.)	H&E↓; leukocyte↓; carbonyl levels↓; sulphhydryl content↑; SOD↑; MDA, ROS↓; IFN-γ, TNF-α, IL-6↓; IL-4, IL-10, TGF-β↑	(90)
Gold	CLPFFD (P12-GNP)	13.0 ± 0.4	−33.5 ± 0.8	1 μM in 50 μL PBS (i.t. pre)	Male C57BL/6 mice -LPS (i.t.)/BMDM	Macrophage targeting, M2 polarization; IL-10↑; iNOS, IL-12, IL-23/40p12, IL-70p6, IL-1↓	(91)
Gold	CLPFFD (P12-GNP)	17.5 ± 0.6	Non	50 pmol per mouse (i.t. pre)	C57BL/6 mice -LPS (i.t.)	H&E↓; Macrophage targeting↑; Treg↑; Neutrophils↓	(92)
Gold	CLPFFD (P12-GNP)	26.9 ± 0.8	Non	25 pmol per mouse (i.t. pre)	Male C57BL/6 mice -LPS (i.n.)/THP-1-Xblue and THP1-Blue ISG	H&E↓; W/D↓; Macrophage targeting↑; BAL protein↓; CCL2, CCL-4↓; cytokine↓; Neutrophils↓	(93)
Se	Se@SiO_2_ NPs	55	Non	1 mg/kg (i.p.)	Male SD rat-paraquat (i.g.)/RLE-6TN	H&E↓; W/D↓; ROS↓; cell viability↑; GSH, SOD↑; MDA↓; IL-1β, TNF-α↓	(94)
Se	Se@SiO_2_ NPs	55	−19.0	100 μg/kg B.W. (i.n. pre)	Male C57BL/6 mice -LPS (i.n.)/Beas-2B	H&E↓; W/D↓; cell viability↑; BAL protein↓; CCL-2, IL-6, IL-1β↓; Macrophage, Neutrophils↓	(95)
Ce	CeO_2_ NPs	220 ± 5	−4.5	4 mg/kg (i.p.)	Male Wistar rat -LPS (i.p.)	H&E↓; ROS↓; TNF-α, CCL-2, IL-6↓; oxygen consumption↑	(96)

Nanomedicine has shown many benefits and potential in the treatment of ARDS. Nanocarriers can improve the activity and stability of enzymes, peptides and nucleic acids as well as their solubility, preventing them from affecting the therapeutic effect in the body due to instability, short half-life and low solubility. For some insoluble drugs, they can also be smoothly delivered to the lungs via nanocarriers to exert their effects. Nanocarriers can significantly change the distribution of drugs in the body, and nano-encapsulated drugs accumulate more in lung tissues by a factor of two or more. It is worth mentioning that the inflamed microenvironment has a large number of aberrantly expressed molecules, which may help nanocarriers to target inflamed lung tissues more precisely. The non-targeted distribution of many drugs leads to the occurrence of side effects that exacerbate the progression of the patient’s disease, and clinical applications are limited. More precise targeted delivery of nanocarriers reduces the biodistribution of drugs in non-target organs and improves drug efficacy (97). The coupling of nanodelivery carriers with multiple nucleic acids (cDNA, siRNA, miRNA) or drugs can exert unexpected effects. This combination therapy strategy in more focused on DEX. DEX coupled to polyamidoamine-delivered lipocalin genes alleviated lung inflammation (98). Some natural actives known to combat ARDS such as curcumin and resveratrol coupled with lipid nano- or polymers as well as therapeutic nucleic acids form complexes that improve nucleic acid delivery efficiency, which may be attributed to their synergistic effect in combating inflammation (99, 100). A ternary complex consisting of plasmid DNA (HO-1), lipopolysaccharide peptide and deoxycholic acid-conjugated polyethylene diamine (PEI-DA) was transfected more efficiently than the binary complex, inducing more HO-1 expression (101). Cationic liposomes encapsulated with various antioxidants N-acetyl cysteine(NAC), vitamin C and E exhibited more potent antioxidant properties (97). Gene-laden nanocarrier complexes may enable more precise and efficient drug delivery by increasing drug solubility as well as enhancing cellular uptake of nucleic acids. It is worth mentioning that the efficacy of nanoparticle drugs is also affected by the mode of administration, intervention time, and physicochemical properties such as size, shape, surface charge, and hydrophilicity (97). This also means that the nanocarriers can optimize the properties according to the drug characteristics and help the drug to be effective to the greatest extent possible. In this regard, the nanodelivery system has more potential for the treatment of ARDS and may be an important strategy for future clinical treatment.

## 4 Conclusions and perspectives

It has been nearly 60 years since the discovery of ARDS, and over the years we have gained a deeper understanding of the causes and pathogenic mechanisms of ARDS, and have made great progress in the treatment of ARDS. The pathological process of ARDS involves alveolar epithelial and endothelial damage, cytokine storms, oxidative stress, and immune dysregulation, which has guided the development of targeted therapies. Anti-inflammatory agents, such as JAK inhibitors and IL-6 inhibitors, have shown potential, particularly when used in early interventions. Additionally, antioxidants like N-acetylcysteine and vitamin C have gained attention, and their therapeutic efficacy has been significantly enhanced through integration with nanotechnology to improve drug targeting. Nanodelivery systems, including liposomes, polymer nanoparticles, and inorganic nanoparticles, offer great potential for ARDS treatment by enhancing drug deposition in the lungs and enabling pH-responsive release to minimize systemic side effects. MSC therapy has also emerged as a promising approach due to its immunomodulatory and tissue repair capabilities, with nano-based delivery systems further improving its stability and targeting. Moreover, personalized treatment strategies tailored to different stages of ARDS have become increasingly important, with future research focusing on phase-specific interventions to better address disease progression under varying triggers. These advancements offer new hope for ARDS treatment and contribute to the development of precision medicine, though further clinical trials are essential to validate the safety and efficacy of these emerging therapies.

The main therapeutic drugs in the clinic are mainly steroids, vitamin C, heparin, and some target-specific inhibitors, which have some effect on the improvement of the disease, but unfortunately, they are all supportive drugs. Nanomaterials have been shown to improve the targeting of delivered drugs, alter their pharmacokinetics and drug distribution, and improve drug efficacy. Although nanodelivery systems have amply demonstrated great potential for the treatment of ARDS, there are still challenges in clinical application. Nano-delivery systems have some off-target effects, and nano-formulations are usually complexes of multiple drugs, which increases the complexity of manufacturing and quality control. This means that further optimization of nano-delivery systems for the treatment of ARDS is needed such as improving chemical stability, particle agglomeration, etc., to enhance the drug delivery efficiency (102). It is worth mentioning that the dose is crucial for the therapeutic efficacy of ARDS. Adverse side effects are often associated with high dose and frequency of drug administration. Therefore, larger patient cohorts are needed to further define the optimal dosage and frequency of use of nanomaterials for the treatment of ARDS in the clinic, and to validate the safety and efficacy of the treatment. Besides, researchers should also focus on continuously exploring the development of more precise therapies based on different stages of ARDS development or different triggers, in order to driving personalized medicine for patients and provide more ideas and rationale for the treatment of ARDS.

## References

[1] Meyer NJ Gattinoni L Calfee CS. Acute respiratory distress syndrome. *Lancet.* (2021) 398:622–37. 34217425 10.1016/S0140-6736(21)00439-6 PMC8248927

[2] Bellani G Laffey JG Pham T Fan E Brochard L Esteban A Epidemiology, patterns of care, and mortality for patients with acute respiratory distress syndrome in intensive care units in 50 countries. *JAMA.* (2016) 315:788–800. 26903337 10.1001/jama.2016.0291

[3] Matthay MA Zemans RL Zimmerman GA Arabi YM Beitler JR Mercat A Acute respiratory distress syndrome. *Nat Rev Dis Primers.* (2019) 5:18. 10.1038/s41572-019-0069-0 PMC6709677 30872586

[4] Yadav H Thompson BT Gajic O. Fifty years of research in ARDS. is acute respiratory distress syndrome a preventable disease? *Am J Respir Crit Care Med.* (2017) 195:725–36. 28040987 10.1164/rccm.201609-1767CI

[5] Bos LD Ware LB. Acute respiratory distress syndrome: causes, pathophysiology, and phenotypes. *Lancet.* (2022) 400:1145–56. 36070787 10.1016/S0140-6736(22)01485-4

[6] Force AD Ranieri VM Rubenfeld GD Thompson BT Ferguson ND Caldwell E Acute respiratory distress syndrome: the Berlin definition. *JAMA.* (2012) 307:2526–33. 22797452 10.1001/jama.2012.5669

[7] Schouten LR Veltkamp F Bos AP van Woensel JB Serpa Neto A Schultz MJ Incidence and mortality of acute respiratory distress syndrome in children: a systematic review and meta-analysis. *Crit Care Med.* (2016) 44:819–29. 26509320 10.1097/CCM.0000000000001388

[8] Khemani RG Smith L Lopez-Fernandez YM Kwok J Morzov R Klein MJ Paediatric acute respiratory distress syndrome incidence and epidemiology (PARDIE): an international, observational study. *Lancet Respir Med.* (2019) 7:115–28. 30361119 10.1016/S2213-2600(18)30344-8 PMC7045907

[9] Kuperminc E Heming N Carlos M Annane D. Corticosteroids in ARDS. *J Clin Med.* (2023) 12:3340. 10.3390/jcm12093340 PMC10179626 37176780

[10] Chaudhuri D Sasaki K Karkar A Sharif S Lewis K Mammen MJ Corticosteroids in COVID-19 and non-COVID-19 ARDS: a systematic review and meta-analysis. *Intens Care Med.* (2021) 47:521–37. 10.1007/s00134-021-06394-2 PMC8054852 33876268

[11] Villar J Ferrando C Martínez D Ambrós A Muñoz T Soler JA Dexamethasone treatment for the acute respiratory distress syndrome: a multicentre, randomised controlled trial. *Lancet Respir Med.* (2020) 8:267–76. 32043986 10.1016/S2213-2600(19)30417-5

[12] Lamouche-Wilquin P Souchard J Pere M Raymond M Asfar P Darreau C Early steroids and ventilator-associated pneumonia in COVID-19-related ARDS. *Crit Care.* (2022) 26:233. 10.1186/s13054-022-04097-8 35918776 PMC9344449

[13] Liu J Zhang S Dong X Li Z Xu Q Feng H Corticosteroid treatment in severe COVID-19 patients with acute respiratory distress syndrome. *J Clin Invest.* (2020) 130:6417–28. 33141117 10.1172/JCI140617 PMC7685724

[14] WHO Rapid Evidence Appraisal for COVID-19 Therapies (REACT) Working Group, Sterne J Murthy S Diaz J Slutsky A Villar J Association between administration of systemic corticosteroids and mortality among critically ill patients with COVID-19: a meta-analysis. *JAMA.* (2020) 324:1330–41. 32876694 10.1001/jama.2020.17023 PMC7489434

[15] Miranda-Massari JR Toro AP Loh D Rodriguez JR Borges RM Marcial-Vega V The effects of vitamin C on the multiple pathophysiological stages of COVID-19. *Life (Basel).* (2021) 11:1341. 10.3390/life11121341 34947872 PMC8708699

[16] Majidi N Rabbani F Gholami S Gholamalizadeh M BourBour F Rastgoo S The effect of vitamin C on pathological parameters and survival duration of critically ill Coronavirus disease 2019 patients: a randomized clinical trial. *Front Immunol.* (2021) 12:717816. 10.3389/fimmu.2021.717816 34975830 PMC8714637

[17] Alamdari DH Moghaddam AB Amini S Keramati MR Zarmehri AM Alamdari AH Application of methylene blue -vitamin C -N-acetyl cysteine for treatment of critically ill COVID-19 patients, report of a phase-I clinical trial. *Eur J Pharmacol.* (2020) 885:173494. 10.1016/j.ejphar.2020.173494 32828741 PMC7440159

[18] Chen L Hu C Hood M Zhang X Zhang L Kan J A novel combination of vitamin C, curcumin and glycyrrhizic acid potentially regulates immune and inflammatory response associated with coronavirus infections: a perspective from system biology analysis. *Nutrients.* (2020) 12:1193. 10.3390/nu12041193 32344708 PMC7230237

[19] Sokary S Ouagueni A Ganji V. Intravenous ascorbic acid and lung function in severely ill COVID-19 patients. *Metabolites.* (2022) 12:865. 10.3390/metabo12090865 PMC9505837 36144269

[20] Fowler AA III Truwit JD Hite RD Morris PE DeWilde C Priday A Effect of vitamin C infusion on organ failure and biomarkers of inflammation and vascular injury in patients with sepsis and severe acute respiratory failure: the CITRIS-ALI randomized clinical trial. *JAMA.* (2019) 322:1261–70. 10.1001/jama.2019.11825 31573637 PMC6777268

[21] Gaspari V Zengarini C Greco S Vangeli V Mastroianni A. Side effects of ruxolitinib in patients with SARS-CoV-2 infection: two case reports. *Int J Antimicrob Agents.* (2020) 56:106023. 10.1016/j.ijantimicag.2020.106023 PMC7243754 32450201

[22] Mechineni A Kassab H Manickam R. Remdesivir for the treatment of COVID 19: review of the pharmacological properties, safety and clinical effectiveness. *Expert Opin Drug Saf.* (2021) 20:1299–307. 34338121 10.1080/14740338.2021.1962284

[23] Shrestha DB Budhathoki P Syed NI Rawal E Raut S Khadka S. Remdesivir: a potential game-changer or just a myth? A systematic review and meta-analysis. *Life Sci.* (2021) 264:118663. 10.1016/j.lfs.2020.118663 33121991 PMC7586105

[24] Izcovich A Siemieniuk RA Bartoszko JJ Ge L Zeraatkar D Kum E Adverse effects of remdesivir, hydroxychloroquine and lopinavir/ritonavir when used for COVID-19: systematic review and meta-analysis of randomised trials. *BMJ Open.* (2022) 12:e048502. 10.1136/bmjopen-2020-048502 PMC8895418 35236729

[25] Huet T Beaussier H Voisin O Jouveshomme S Dauriat G Lazareth I Anakinra for severe forms of COVID-19: a cohort study. *Lancet Rheumatol.* (2020) 2:e393–400. 32835245 10.1016/S2665-9913(20)30164-8 PMC7259909

[26] Balkhair A Al-Zakwani I Al Busaidi M Al-Khirbash A Al Mubaihsi S BaTaher H Anakinra in hospitalized patients with severe COVID-19 pneumonia requiring oxygen therapy: results of a prospective, open-label, interventional study. *Int J Infect Dis.* (2021) 103:288–96. 10.1016/j.ijid.2020.11.149 33217576 PMC7670920

[27] Gupta B Ahluwalia P Gupta N Gupta A. Role of nebulized heparin in clinical outcome of COVID-19 patients with respiratory symptoms: a systematic review. *Indian J Crit Care Med.* (2023) 27:572–9. 10.5005/jp-journals-10071-24511 37636853 PMC10452767

[28] Dixon B Smith RJ Campbell DJ Moran JL Doig GS Rechnitzer T Nebulised heparin for patients with or at risk of acute respiratory distress syndrome: a multicentre, randomised, double-blind, placebo-controlled phase 3 trial. *Lancet Respir Med.* (2021) 9:360–72. 33493448 10.1016/S2213-2600(20)30470-7 PMC7826120

[29] Mi P. Stimuli-responsive nanocarriers for drug delivery, tumor imaging, therapy and theranostics. *Theranostics.* (2020) 10:4557–88. 32292515 10.7150/thno.38069 PMC7150471

[30] van Rijt SH Bein T Meiners S. Medical nanoparticles for next generation drug delivery to the lungs. *Eur Respir J.* (2014) 44:765–74. 10.1183/09031936.00212813 24791828

[31] Fernandez Tena A Casan Clara P. Deposition of inhaled particles in the lungs. *Arch Bronconeumol.* (2012) 48:240–6. 22464044 10.1016/j.arbres.2012.02.003

[32] Muhammad W Zhai Z Wang S Gao C. Inflammation-modulating nanoparticles for pneumonia therapy. *Wiley Interdiscip Rev Nanomed Nanobiotechnol.* (2022) 14:e1763. 10.1002/wnan.1763 34713969

[33] Roy I Vij N. Nanodelivery in airway diseases: challenges and therapeutic applications. *Nanomedicine.* (2010) 6:237–44. 19616124 10.1016/j.nano.2009.07.001 PMC2847663

[34] Yacobi NR Demaio L Xie J Hamm-Alvarez SF Borok Z Kim KJ Polystyrene nanoparticle trafficking across alveolar epithelium. *Nanomedicine.* (2008) 4:139–45. 18375191 10.1016/j.nano.2008.02.002

[35] Arber Raviv S Alyan M Egorov E Zano A Harush MY Pieters C Lung targeted liposomes for treating ARDS. *J Control Release.* (2022) 346:421–33. 35358610 10.1016/j.jconrel.2022.03.028 PMC8958843

[36] Pooladanda V Thatikonda S Sunnapu O Tiwary S Vemula PK Talluri M iRGD conjugated nimbolide liposomes protect against endotoxin induced acute respiratory distress syndrome. *Nanomedicine.* (2021) 33:102351. 10.1016/j.nano.2020.102351 33418136 PMC7833751

[37] Jiang L Guo P Ju J Zhu X Wu S Dai J. Inhalation of L-arginine-modified liposomes targeting M1 macrophages to enhance curcumin therapeutic efficacy in ALI. *Eur J Pharm Biopharm.* (2023) 182:21–31. 10.1016/j.ejpb.2022.11.017 36442537

[38] Chono S Tanino T Seki T Morimoto K. Efficient drug targeting to rat alveolar macrophages by pulmonary administration of ciprofloxacin incorporated into mannosylated liposomes for treatment of respiratory intracellular parasitic infections. *J Control Release.* (2008) 127:50–8. 10.1016/j.jconrel.2007.12.011 18230410

[39] Chen XY Wang SM Li N Hu Y Zhang Y Xu JF Creation of lung-targeted dexamethasone immunoliposome and its therapeutic effect on bleomycin-induced lung injury in rats. *PLoS One.* (2013) 8:e58275. 10.1371/journal.pone.0058275 23516459 PMC3597622

[40] Huang Z Wang H Long J Lu Z Chun C Li X. Neutrophil membrane-coated therapeutic liposomes for targeted treatment in acute lung injury. *Int J Pharm.* (2022) 624:121971. 10.1016/j.ijpharm.2022.121971 35787461 PMC9365401

[41] Kardara M Hatziantoniou S Sfika A Vassiliou AG Mourelatou E Muagkou C Caveolar uptake and endothelial-protective effects of nanostructured lipid carriers in acid aspiration murine acute lung injury. *Pharm Res.* (2013) 30:1836–47. 10.1007/s11095-013-1027-2 23549752

[42] Luo J Li X Dong S Zhu P Liu W Zhang S Layer-by-layer coated hybrid nanoparticles with pH-sensitivity for drug delivery to treat acute lung infection. *Drug Deliv.* (2021) 28:2460–8. 10.1080/10717544.2021.2000676 34766544 PMC8592614

[43] Liu C Xi L Liu Y Mak JC Mao S Wang Z An inhalable hybrid biomimetic nanoplatform for sequential drug release and remodeling lung immune homeostasis in acute lung injury treatment. *ACS Nano.* (2023) 17:11626–44. 10.1021/acsnano.3c02075 37285229

[44] Kusumoto K Akita H Ishitsuka T Matsumoto Y Nomoto T Furukawa R Lipid envelope-type nanoparticle incorporating a multifunctional peptide for systemic siRNA delivery to the pulmonary endothelium. *ACS Nano.* (2013) 7:7534–41. 10.1021/nn401317t 23909689

[45] Wijagkanalan W Kawakami S Higuchi Y Yamashita F Hashida M. Intratracheally instilled mannosylated cationic liposome/NFkappaB decoy complexes for effective prevention of LPS-induced lung inflammation. *J Control Release.* (2011) 149:42–50. 10.1016/j.jconrel.2009.12.016 20035809

[46] Wijagkanalan W Higuchi Y Kawakami S Teshima M Sasaki H Hashida M. Enhanced anti-inflammation of inhaled dexamethasone palmitate using mannosylated liposomes in an endotoxin-induced lung inflammation model. *Mol Pharmacol.* (2008) 74:1183–92. 10.1124/mol.108.050153 18669445

[47] Fei Q Shalosky EM Barnes R Shukla VC Xu S Ballinger MN Macrophage-targeted lipid nanoparticle delivery of microRNA-146a to mitigate hemorrhagic shock-induced acute respiratory distress syndrome. *ACS Nano.* (2023) 17:16539–52. 37595605 10.1021/acsnano.3c01814 PMC10754353

[48] Qiao Q Liu X Cui K Li X Tian T Yu Y Hybrid biomimetic nanovesicles to drive high lung biodistribution and prevent cytokine storm for ARDS treatment. *ACS Nano.* (2022) 16:15124–40. 10.1021/acsnano.2c06357 36037505

[49] Li N Weng D Wang SM Zhang Y Chen SS Yin ZF Surfactant protein-A nanobody-conjugated liposomes loaded with methylprednisolone increase lung-targeting specificity and therapeutic effect for acute lung injury. *Drug Deliv.* (2017) 24:1770–81. 10.1080/10717544.2017.1402217 29160134 PMC8241200

[50] Li S Chen L Wang G Xu L Hou S Chen Z Anti-ICAM-1 antibody-modified nanostructured lipid carriers: a pulmonary vascular endothelium-targeted device for acute lung injury therapy. *J Nanobiotechnol.* (2018) 16:105. 10.1186/s12951-018-0431-5 30594254 PMC6311082

[51] Li SJ Wang XJ Hu JB Kang XQ Chen L Xu XL Targeting delivery of simvastatin using ICAM-1 antibody-conjugated nanostructured lipid carriers for acute lung injury therapy. *Drug Deliv.* (2017) 24:402–13. 28165814 10.1080/10717544.2016.1259369 PMC8248938

[52] Zhou A Chen K Gao Y Zhou X Tian Z Chen W Bioengineered neutrophil extinguisher targets cascade immune pathways of macrophages for alleviating cytokine storm in pneumonia. *ACS Nano.* (2023) 17:16461–77. 10.1021/acsnano.3c00227 37596997

[53] Park HH Park W Lee YY Kim H Seo HS Choi DW Bioinspired DNase-I-coated melanin-like nanospheres for modulation of infection-associated NETosis dysregulation. *Adv Sci (Weinh).* (2020) 7:2001940. 10.1002/advs.202001940 PMC7645930 33173718

[54] Jiang J Huang K Xu S Garcia JG Wang C Cai H. Erratum to targeting NOX4 alleviates sepsis-induced acute lung injury via attenuation of redox-sensitive activation of CaMKII/ERK1/2/MLCK and endothelial cell barrier dysfunction, Redox Biology 36 (2020) 101638. *Redox Biol.* (2021) 48:102200. 10.1016/j.redox.2020.101638 34844898 PMC8710995

[55] Huppert LA Matthay MA Ware LB. Pathogenesis of acute respiratory distress syndrome. *Semin Respir Crit Care Med.* (2019) 40:31–9. 31060086 10.1055/s-0039-1683996 PMC7060969

[56] Li W Li D Chen Y Abudou H Wang H Cai J Classic signaling pathways in alveolar injury and repair involved in sepsis-induced ALI/ARDS: new research progress and prospect. *Dis Markers.* (2022) 2022:6362344. 10.1155/2022/6362344 35726235 PMC9206211

[57] Deshpande PP Biswas S Torchilin VP. Current trends in the use of liposomes for tumor targeting. *Nanomedicine (Lond).* (2013) 8:1509–28. 23914966 10.2217/nnm.13.118 PMC3842602

[58] Pooladanda V Thatikonda S Muvvala SP Devabattula G Godugu C. BRD4 targeting nanotherapy prevents lipopolysaccharide induced acute respiratory distress syndrome. *Int J Pharm.* (2021) 601:120536. 10.1016/j.ijpharm.2021.120536 33781885 PMC7997899

[59] Xu Q Guo M Jin X Jin Q He Z Xiao W Interferon regulatory factor 5 siRNA-loaded folate-modified cationic liposomes for acute lung injury therapy. *J Biomed Nanotechnol.* (2021) 17:466–76. 10.1166/jbn.2021.3046 33875081

[60] Dong J Liao W Tan LH Yong A Peh WY Wong WS. Gene silencing of receptor-interacting protein 2 protects against cigarette smoke-induced acute lung injury. *Pharmacol Res.* (2019) 139:560–8. 10.1016/j.phrs.2018.10.016 30394320

[61] Zoulikha M Xiao Q Boafo GF Sallam MA Chen Z He W. Pulmonary delivery of siRNA against acute lung injury/acute respiratory distress syndrome. *Acta Pharm Sin B.* (2022) 12:600–20. 10.1016/j.apsb.2021.08.009 34401226 PMC8359643

[62] Yu HP Liu FC Lin CY Umoro A Trousil J Hwang TL Suppression of neutrophilic inflammation can be modulated by the droplet size of anti-inflammatory nanoemulsions. *Nanomedicine (Lond).* (2020) 15:773–91. 10.2217/nnm-2019-0407 32193978

[63] Jiang S Li S Hu J Xu X Wang X Kang X Combined delivery of angiopoietin-1 gene and simvastatin mediated by anti-intercellular adhesion molecule-1 antibody-conjugated ternary nanoparticles for acute lung injury therapy. *Nanomedicine.* (2019) 15:25–36. 10.1016/j.nano.2018.08.009 30193816

[64] Bian S Cai H Cui Y Liu W Xiao C. Nanomedicine-based therapeutics to combat acute lung injury. *Int J Nanomed.* (2021) 16:2247–69. 10.2147/IJN.S300594 33776431 PMC7987274

[65] Sen K Mandal M. Second generation liposomal cancer therapeutics: transition from laboratory to clinic. *Int J Pharm.* (2013) 448:28–43. 23500602 10.1016/j.ijpharm.2013.03.006

[66] van Etten EW ten Kate MT Snijders SV Bakker-Woudenberg IA. Administration of liposomal agents and blood clearance capacity of the mononuclear phagocyte system. *Antimicrob Agents Chemother.* (1998) 42:1677–81. 9661003 10.1128/aac.42.7.1677 PMC105665

[67] Newman SP. Drug delivery to the lungs: challenges and opportunities. *Ther Deliv.* (2017) 8:647–61. 10.4155/tde-2017-0037 28730933

[68] Hald Albertsen C Kulkarni JA Witzigmann D Lind M Petersson K Simonsen JB. The role of lipid components in lipid nanoparticles for vaccines and gene therapy. *Adv Drug Deliv Rev.* (2022) 188:114416. 10.1016/j.addr.2022.114416 PMC9250827 35787388

[69] Hou X Zaks T Langer R Dong Y. Lipid nanoparticles for mRNA delivery. *Nat Rev Mater.* (2021) 6:1078–94. 34394960 10.1038/s41578-021-00358-0 PMC8353930

[70] Jung HN Lee SY Lee S Youn H Im HJ. Lipid nanoparticles for delivery of RNA therapeutics: current status and the role of *in vivo* imaging. *Theranostics.* (2022) 12:7509–31. 10.7150/thno.77259 36438494 PMC9691360

[71] Severino P Andreani T Macedo AS Fangueiro JF Santana MH Silva A Current state-of-art and new trends on lipid nanoparticles (SLN and NLC) for oral drug delivery. *J Drug Deliv.* (2012) 2012:750891. 10.1155/2012/750891 22175030 PMC3228282

[72] Jin F Liu D Yu H Qi J You Y Xu X Sialic acid-functionalized PEG-PLGA microspheres loading mitochondrial-targeting-modified curcumin for acute lung injury therapy. *Mol Pharm.* (2019) 16:71–85. 10.1021/acs.molpharmaceut.8b00861 30431285

[73] Attias Cohen S Kingma PS Whitsett JA Goldbart R Traitel T Kost J. SP-D loaded PLGA nanoparticles as drug delivery system for prevention and treatment of premature infant’s lung diseases. *Int J Pharm.* (2020) 585:119387. 10.1016/j.ijpharm.2020.119387 32473376

[74] Jin H Zhao Z Lan Q Zhou H Mai Z Wang Y Nasal delivery of hesperidin/chitosan nanoparticles suppresses cytokine storm syndrome in a mouse model of acute lung injury. *Front Pharmacol.* (2020) 11:592238. 10.3389/fphar.2020.592238 33584267 PMC7873598

[75] Villa-Hermosilla MC Negro S Barcia E Hurtado C Montejo C Alonso M Celecoxib microparticles for inhalation in COVID-19-related acute respiratory distress syndrome. *Pharmaceutics.* (2022) 14:1392. 10.3390/pharmaceutics14071392 35890288 PMC9320401

[76] Moshiri M Mehmannavaz F Hashemi M Yazdian-Robati R Shabazi N Etemad L. Evaluation of the efficiency of simvastatin loaded PLGA nanoparticles against acute paraquat-intoxicated rats. *Eur J Pharm Sci.* (2022) 168:106053. 10.1016/j.ejps.2021.106053 34728365

[77] Kotta S Aldawsari HM Badr-Eldin SM Binmahfouz LS Bakhaidar RB Sreeharsha N Lung targeted lipopolymeric microspheres of dexamethasone for the treatment of ARDS. *Pharmaceutics.* (2021) 13:1347. 10.3390/pharmaceutics13091347 34575422 PMC8471313

[78] Zhai Z Ouyang W Yao Y Zhang Y Zhang H Xu F Dexamethasone-loaded ROS-responsive poly(thioketal) nanoparticles suppress inflammation and oxidative stress of acute lung injury. *Bioact Mater.* (2022) 14:430–42. 10.1016/j.bioactmat.2022.01.047 35415281 PMC8965854

[79] Su M Yang B Xi M Qiang C Yin Z. Therapeutic effect of pH-Responsive dexamethasone prodrug nanoparticles on acute lung injury. *J Drug Deliv Sci Technol.* (2021) 66:102738. 10.1016/j.jddst.2021.102738 36568326 PMC9760482

[80] Ding Y Lv B Zheng J Lu C Liu J Lei Y RBC-hitchhiking chitosan nanoparticles loading methylprednisolone for lung-targeting delivery. *J Control Release.* (2022) 341:702–15. 10.1016/j.jconrel.2021.12.018 34933051 PMC8684098

[81] Zhang CY Lin W Gao J Shi X Davaritouchaee M Nielsen AE pH-responsive nanoparticles targeted to lungs for improved therapy of acute lung inflammation/injury. *ACS Appl Mater Interfaces.* (2019) 11:16380–90. 30973702 10.1021/acsami.9b04051 PMC6542597

[82] Almatroudi A Alsahli MA Syed MA Khan AA Rahmani AH. Regulation of pro-inflammatory macrophage polarization via lipid nanoparticles mediated delivery of anti-prostaglandin-E2 siRNA. *Curr Issues Mol Biol.* (2022) 45:1–11. 10.3390/cimb45010001 36661487 PMC9856913

[83] Lee YY Park HH Park W Kim H Jang JG Hong KS Long-acting nanoparticulate DNase-1 for effective suppression of SARS-CoV-2-mediated neutrophil activities and cytokine storm. *Biomaterials.* (2021) 267:120389. 10.1016/j.biomaterials.2020.120389 33130319 PMC7583619

[84] Fesenmeier DJ Suresh MV Kim S Park S Raghavendran K Won YY. Polymer lung surfactants attenuate direct lung injury in mice. *ACS Biomater Sci Eng.* (2023) 9:2716–30. 10.1021/acsbiomaterials.3c00061 37079432 PMC11973738

[85] Kim S Fesenmeier DJ Park S Torregrosa-Allen SE Elzey BD Won YY. Pulmonary pharmacokinetics of polymer lung surfactants following pharyngeal administration in mice. *Biomacromolecules.* (2022) 23:2471–84. 10.1021/acs.biomac.2c00221 35580262 PMC12001817

[86] Kim HC Suresh MV Singh VV Arick DQ Machado-Aranda DA Raghavendran K Polymer lung surfactants. *ACS Appl Bio Mater.* (2018) 1:581–92. 10.1021/acsabm.8b00061 PMC6322699 30627707

[87] D’Almeida AP Pacheco de Oliveira MT de Souza ET de Sa Coutinho D Ciambarella BT Gomes CR alpha-bisabolol-loaded lipid-core nanocapsules reduce lipopolysaccharide-induced pulmonary inflammation in mice. *Int J Nanomed.* (2017) 12:4479–91. 10.2147/IJN.S130798 28684908 PMC5484570

[88] de Oliveira MT de Sa Coutinho D Tenorio de Souza E Staniscuaski Guterres S Pohlmann AR Silva PM Orally delivered resveratrol-loaded lipid-core nanocapsules ameliorate LPS-induced acute lung injury via the ERK and PI3K/Akt pathways. *Int J Nanomed.* (2019) 14:5215–28. 10.2147/IJN.S200666 31371957 PMC6636190

[89] Jeon T Luther DC Goswami R Bell C Nagaraj H Cicek YA Engineered polymer-siRNA polyplexes provide effective treatment of lung inflammation. *ACS Nano.* (2023) 17:4315–26. 10.1021/acsnano.2c08690 36802503 PMC10627429

[90] Casagrande LR Porto GD Colares MC Venturini LM Silveira GB Mendes C Green synthesis of gold nanoparticles modulates lipopolysaccharide-induced lung inflammation in Wistar rats. *Basic Clin Pharmacol Toxicol.* (2023) 132:473–85. 10.1111/bcpt.13854 36882317

[91] Wang L Zhang H Sun L Gao W Xiong Y Ma A Manipulation of macrophage polarization by peptide-coated gold nanoparticles and its protective effects on acute lung injury. *J Nanobiotechnol.* (2020) 18:38. 10.1186/s12951-020-00593-7 32101146 PMC7045427

[92] Xiong Y Gao W Xia F Sun Y Sun L Wang L Peptide-gold nanoparticle hybrids as promising anti-inflammatory nanotherapeutics for acute lung injury: *in vivo* efficacy, biodistribution, and clearance. *Adv Healthc Mater.* (2018) 7:e1800510. 10.1002/adhm.201800510 30101578

[93] Gao W Wang Y Xiong Y Sun L Wang L Wang K Size-dependent anti-inflammatory activity of a peptide-gold nanoparticle hybrid *in vitro* and in a mouse model of acute lung injury. *Acta Biomater.* (2019) 85:203–17. 10.1016/j.actbio.2018.12.046 30597258 PMC8960115

[94] Zhu Y Deng G Ji A Yao J Meng X Wang J Porous Se@SiO(2) nanospheres treated paraquat-induced acute lung injury by resisting oxidative stress. *Int J Nanomedicine.* (2017) 12:7143–52. 10.2147/IJN.S143192 29026307 PMC5627737

[95] Wang M Wang K Deng G Liu X Wu X Hu H Mitochondria-modulating porous Se@SiO(2) nanoparticles provide resistance to oxidative injury in airway epithelial cells: implications for acute lung injury. *Int J Nanomed.* (2020) 15:2287–302. 10.2147/IJN.S240301 32280221 PMC7127826

[96] Serebrovska Z Swanson RJ Portnichenko V Shysh A Pavlovich S Tumanovska L Anti-inflammatory and antioxidant effect of cerium dioxide nanoparticles immobilized on the surface of silica nanoparticles in rat experimental pneumonia. *Biomed Pharmacother.* (2017) 92:69–77. 10.1016/j.biopha.2017.05.064 28531802

[97] Qiao Q Liu X Yang T Cui K Kong L Yang C Nanomedicine for acute respiratory distress syndrome: the latest application, targeting strategy, and rational design. *Acta Pharm Sin B.* (2021) 11:3060–91. 10.1016/j.apsb.2021.04.023 33977080 PMC8102084

[98] Piao C Park JH Lee M. Anti-inflammatory therapeutic effect of adiponectin gene delivery using a polymeric carrier in an acute lung injury model. *Pharm Res.* (2017) 34:1517–26. 10.1007/s11095-017-2175-6 28493099

[99] Oh B Lee M. Combined delivery of HMGB-1 box A peptide and S1PLyase siRNA in animal models of acute lung injury. *J Control Release.* (2014) 175:25–35. 10.1016/j.jconrel.2013.12.008 24361371

[100] Park JH Kim HA Park JH Lee M. Amphiphilic peptide carrier for the combined delivery of curcumin and plasmid DNA into the lungs. *Biomaterials.* (2012) 33:6542–50. 10.1016/j.biomaterials.2012.05.046 22687757

[101] Kim JY Piao C Kim G Lee S Lee MS Jeong JH Combined delivery of a lipopolysaccharide-binding peptide and the heme oxygenase-1 gene using deoxycholic acid-conjugated polyethylenimine for the treatment of acute lung injury. *Macromol Biosci.* (2017) 17. 10.1002/mabi.201600490 28508430

[102] Prasanna P Rathee S Upadhyay A Sulakshana S. Nanotherapeutics in the treatment of acute respiratory distress syndrome. *Life Sci.* (2021) 276:119428. 10.1016/j.lfs.2021.119428 PMC7999693 33785346

